# Computational Investigation of Environment-Noise Interaction in Single-Cell Organisms: The Merit of Expression Stochasticity Depends on the Quality of Environmental Fluctuations

**DOI:** 10.1038/s41598-017-17441-8

**Published:** 2018-01-10

**Authors:** Anja Lück, Lukas Klimmasch, Peter Großmann, Sebastian Germerodt, Christoph Kaleta

**Affiliations:** 1 Department of Bioinformatics, Friedrich Schiller University, Jena, 07743 Germany; 2 Research Group Theoretical Systems Biology, Friedrich Schiller University, Jena, 07743 Germany; 3 Research Group Medical Systems Biology, Institute for Experimental Medicine, Christian-Albrechts-University, Kiel, 24105 Germany

## Abstract

Organisms need to adapt to changing environments and they do so by using a broad spectrum of strategies. These strategies include finding the right balance between expressing genes before or when they are needed, and adjusting the degree of noise inherent in gene expression. We investigated the interplay between different nutritional environments and the inhabiting organisms’ metabolic and genetic adaptations by applying an evolutionary algorithm to an agent-based model of a concise bacterial metabolism. Our results show that constant environments and rapidly fluctuating environments produce similar adaptations in the organisms, making the predictability of the environment a major factor in determining optimal adaptation. We show that exploitation of expression noise occurs only in some types of fluctuating environment and is strongly dependent on the quality and availability of nutrients: stochasticity is generally detrimental in fluctuating environments and beneficial only at equal periods of nutrient availability and above a threshold environmental richness. Moreover, depending on the availability and nutritional value of nutrients, nutrient-dependent and stochastic expression are both strategies used to deal with environmental changes. Overall, we comprehensively characterize the interplay between the quality and periodicity of an environment and the resulting optimal deterministic and stochastic regulation strategies of nutrient-catabolizing pathways.

## Introduction

Most organisms face a multitude of potential environments differing in hospitality as well as timescale with respect to changes. This variance in environmental conditions leads to a fundamental problem: How to allocate resources effectively in any environment, so that growth rate and adaptation to changes in the environment are optimally balanced? Organisms need to take into account what is necessary in the present environment and what is potentially needed after a change in environmental conditions. Adaptation through evolution enables anticipation of environmental conditions^1^, but there is always some degree of uncertainty. Organisms have developed a myriad of gene regulatory strategies for dealing with uncertainties in their habitats. Genes can, for example, be expressed constitutively, enabling a fast response but also potentially wasting resources on unnecessary expression, or only after receiving an environmental stimulus, a more resource-efficient approach that entails a slower reaction.

In addition to gene expression in response to an environmental cue, genes can also be expressed randomly^2^ and thus only by a part of the population^3^. Stochasticity or expression noise is an inherent feature of gene expression^4^. A large part of expression noise in bacteria is directly generated by bursty transcription and translation^5–9^. By creating small differences in gene product abundance, expression noise allows genetically identical cells to differ from one another, causing differences in reproductive fitness between individuals that share genotype as well as environment^10^. Stochastic gene expression in itself is not genetic, but the regulatory system that influences its magnitude is^11^: Expression noise is an evolvable characteristic of each gene^12,13^. Selection influences expression stochasticity dependent on the demands of the environment^13,14^ and of gene function^7,13–16^. Expression stochasticity contributes to various regulatory tasks^13,17,18^ such as establishing binary phenotypes that require a decision^19^. Each cell switches its phenotype by itself due to stochasticity in gene expression, and this decision can then be reinforced by gene regulatory mechanisms^2^. This process operates on a shorter timescale than mutation and allows at least a part of the population to survive sudden environmental changes by possessing the right adaptation at the right time^3,20^. It is a bet-hedging strategy that can often be found in fluctuating environments^21^. Noise control is wasteful at the translational level^22^, creating a trade-off between flexibility caused by high noise levels and translational resource efficiency^23^. There are differences in noise levels between functional gene groups^6,24^ and the molecular mechanisms of variability received due attention^13,25–31^, but only little is known about how the environmental conditions themselves impact the optimal level of expression stochasticity^3,20,32,33^.

**Figure 1:**
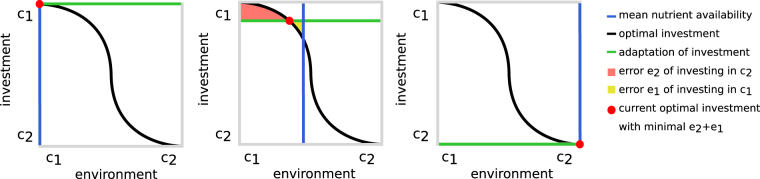
Optimizing adaptation to multistate environments. The environmental state is defined by the availability of nutrients (i.e. *c*_1_ and *c*_2_). The nutrient uptake by a cell determines its fitness. Cells have to decide in which metabolic pathway they invest to increase the influx of *c*_1_ and *c*_2_. The mean state of the environment (blue line), given by the current nutrient availability, is represented by a perpendicular. Cells can adapt to different environmental states (green line). The optimal investment (red dot) is achieved by minimizing errors of type *e*_1_ (red area) and *e*_2_ (yellow area) representing investment into improper uptake paths. The hypothetical optimal linkage between environmental state and the investment ratio of *c*_1_ and *c*_2_ is initially unknown (e.g. here sigmoid) and subject to evolutionary optimization.

The environment of microorganisms switches between different states (see Fig. 1 for a schematic overview). Adapting to one specific state of the environment can entail not being adapted to other possible states. For instance, assuming an environment with two alternative nutritional sources *c*_1_ and *c*_2_, two types of errors can occur: investing in the uptake of resource *c*_2_ while resource *c*_1_ is present (error *e*_1_) and vice versa (error *e*_2_). The optimal investment ratio between *c*_1_ and *c*_2_ of an individual cell is achieved by minimizing the sum of both errors over time. This minimization depends only on the environmental conditions and the individual’s internal state and its past actions. As resource uptake and fitness are tightly linked, each deviation of an individual’s investment ratio from an optimal ratio decreases the individual’s fitness and the frequency of its investment ratio in the population. Thus, uptake rates are subject to selection. Finding a balance between an adaptation that minimizes response times and at the same time maximizes growth rate by minimizing waste of resources is an evolutionary optimization problem. *Escherichia coli*, for example, expresses uptake pathways for alternative substrates even in the absence of the corresponding nutritional sources especially in conditions of nutrient scarcity^34^. It was shown that in dynamic environments, a strategy that minimizes variance in growth rates rather than maximization of growth rates is optimal^35,36^. Recently, the absolute investment into unused functions in *E*. *coli* has been quantified based on an extensive absolute protein abundance dataset. It was found that depending on environmental conditions up to 50% of the proteome was invested into unused proteins and that nutrient- as well as stress-response functions made up a considerable part of this investment^37^. The optimal investment into nutrient-catabolizing pathways, depending on nutrient availability, is affected by many factors, for example by the frequency and amplitude of the resources’ availabilities, different uptake costs and nutritional value of the resources. Individuals may never reach an optimal investment ratio as environmental fluctuations usually occur at a much faster time-scale than evolutionary adaptation.

In this work, we investigated the interplay of environmental resource availability with the adaptation of metabolic regulation, including expression stochasticity. How does the magnitude and impact of stochasticity depend on the exact environmental conditions and what are important factors in deciding in which conditions the utilization of stochasticity is of advantage? To answer these questions, we developed a model containing bacteria with a minimal metabolism that are subjected to evolution through variation and selection in different environmental regimes. This approach allows us to study the interaction between the environment and expression stochasticity, regardless of its origin, and we can show that even in a very concise model of bacterial metabolism complex adaptive states can arise due to environmental fluctuations. Overall, we find that environmental conditions strongly influence the optimal strategy for regulating nutrient-catabolizing pathways. Surprisingly, expression noise is generally detrimental and only of advantage at equal periods of nutrient availability and above a threshold environmental richness.

## Results

### Agent-based model for bacterial metabolism

**Figure 2:**
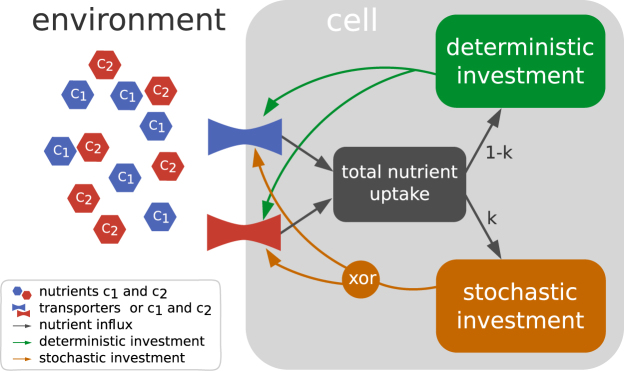
Bacterial agent. Bacteria use transporters *t*_*i*_ to take up nutrients *c*_*i*_ from the environment and invest them into new transporters. Uptake is partitioned between two nutrient-specific pathways. Partitioning is determined using metabolic rates for constitutive and nutrient-dependent investment. A part *k* of the uptake is invested stochastically (all-or-nothing, referred to as “xor”), depending on the metabolic rates, using a separate set of metabolic rates for constitutive and nutrient-dependent investment. Bacteria divide when a threshold size is reached. Daughter cells contain mutated metabolic rates and *k*. Simulations stop when a preset mean number of generations is reached.

We simulated a concise bacterial metabolism comprising constitutive expression, gene expression that is regulated transcriptionally dependent on nutrient availability, and gene expression stochasticity using an agent-based model combined with an evolutionary algorithm. Bacteria took up nutrients from their environment using nutrient-specific transporters. This uptake was partitioned between two pathways that invested in the respective transporters (see Fig. 2). Partitioning depended on the evolvable pathway-specific metabolic rates for nutrient-dependent and constitutive investment. The calculation of relative investment into the transporters of each pathway takes into account the pathway-specific nutrient-dependent metabolic rate if the nutrient is present in the environment and the pathway-specific constitutive metabolic rate irrespective of the nutrient’s presence in the environment. To model noisy expression, an evolvable part *k* of the nutrient uptake was invested stochastically into only one of the pathways. This investment was dependent on a separate set of evolvable pathway-specific nutrient-dependent and constitutive metabolic rates. Upon reaching a threshold size, bacteria divided and the metabolic rates and *k* were mutated based on the rates of the ancestor. A metabolic setup that fit the environment better led to faster growth and more descendants than a less fitting setup. This setup was then more prevalent in the population and less likely to be deleted by regularly occuring dilution events that randomly removed 70 per cent of the population. The simulations stopped when all bacteria were dead or a preset mean number of generations had been reached.

**Figure 3:**
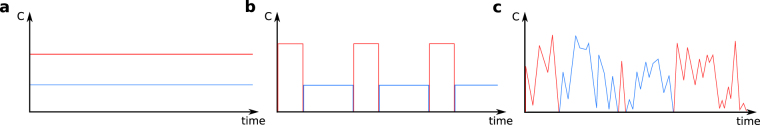
Environmental scenarios. (**a**) Constant environment, both nutrients available in the same concentration at all times. (**b**) Deterministically alternating environment, both nutrients occur alternately, concentration on occurrence and duration of occurrence fixed. (**c**) Randomly alternating environment, both nutrients occur alternately, concentrations and durations of occurrence uniformly distributed.

The environment was specified by the concentrations of the two nutrients and the respective durations of their availability. We tested three environmental scenarios: (i) a constant environment, in which both nutrients were available in the same concentration at all times, (ii) a deterministically alternating environment, in which both nutrients occurred in turns, their concentration and duration of occurrence fixed, and (iii) a randomly alternating environment, in which both nutrients occurred in turns, their concentrations and durations of occurrence uniformly distributed (Fig. 3). When applicable, different combinations of nutrient concentrations and of durations of nutrient occurrence were tested to represent a broad variety of natural habitats of microorganisms. In all cases, 100 repetitions were performed until a mean of 5000 generations had been reached.

We then calculated for each individual the fraction of investment into the pathway of the first nutrient (*c*_1_ investment), the fraction of investment that occurred only in the presence of the respective nutrient in the environment (adaptive investment) and the fraction of investment that was made stochastically (through parameter *k*). We then performed simulation runs in which we prohibited stochastic investment (*k* = 0, “stochasticity is disabled”) to compare to simulations in which stochastic investment could take place (“stochasticity is enabled”).

#### Constant Environments

**Figure 4:**
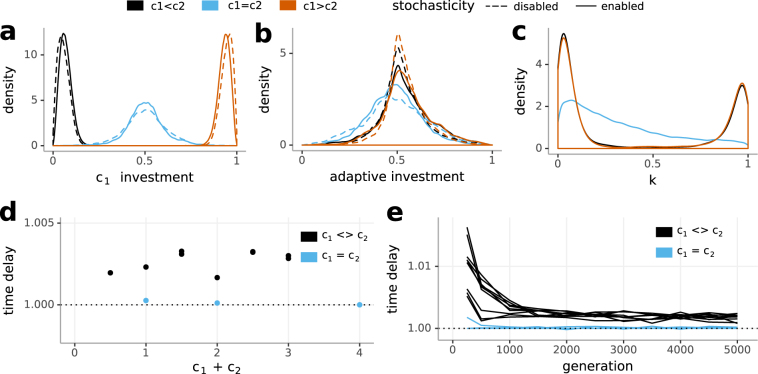
Constant environment: investment strategies and required simulation steps depending on variations in environmental nutrient concentrations. The legend at the top applies to (**a**–**c**). (**a**) Investment occurs into the nutrient available in the higher concentration, (**b**) adaptive and constitutive investment are balanced and (**c**) stochastic investment (*k*) in simulations with stochasticity enabled peaks near 0 and 1 if nutrients are not available in the same concentration. (**d**) The plot shows the ratio of mean update steps between simulations with stochasticity enabled and simulations with stochasticity disabled (*time delay*). Simulations with stochasticity enabled always required more update steps to reach 5000 generations. (**e**) This time delay decreases as generations increase.

In a constant environment, investment mainly occurred into the pathway of the nutrient available in the higher concentration (Fig. 4a). Equal nutrient availability lead to equal investment. Adaptive investment approached values near 0.5 (Fig. 4b). Stochastic investment (*k*) peaked near 0 and 1 if nutrient concentrations were unequal (Fig. 4c). By using mainly deterministic investment (peak near 0), stochasticity was genetically switched off; by using mainly stochastic investment (peak near 1), the same was true: The probability of investment into either nutrient pathway was determined by the metabolic rates. As investment mainly went into the pathway of the nutrient available in the higher concentration, the probability of investment into the other pathway was very low. When nutrient concentrations were equal, we found a broader distribution of both adaptive and stochastic investment, which indicates weaker selection pressure on finding an optimum ratio.

The mean amount of required update steps can be used as a proxy for the advantage of stochasticity. To this end, we calculated the ratio of mean update steps between simulations with stochasticity enabled and simulations with stochasticity disabled (*time delay*). Simulations in the constant environment always required significantly more update steps when the stochastic pathway was switched on (Fig. 4d, Supplementary Table), showing a detrimental effect of stochasticity, except at high and equal nutrient concentrations. This detrimental effect could either manifest as a general reduction in growth rates or a reduction in the rate of adaptation. In the former case, we would expect a constant ratio independent of generations reached whereas in the latter case we would expect that the ratio reduces with increasing generation count due to the population evolving to an optimum, but at different speeds. Figure 4e shows a decrease of the time delay across generations, indicating a reduced speed of adaptation due to the presence of stochasticity, and no general difference in growth rates once the population had evolved to its optimum in the corresponding environment.

#### Deterministically Alternating Environments

**Figure 5:**
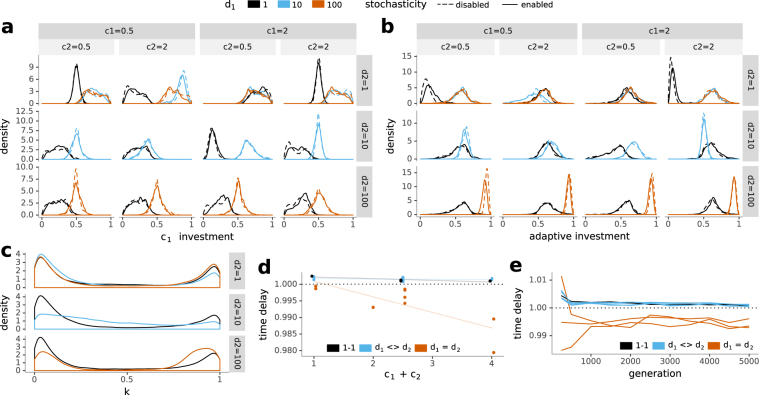
Deterministically alternating environment: investment strategies and required simulation steps as they depend on variations in environmental nutient concentrations und duration of availability. The legend at the top applies to (**a**–**c**). (**a**) The influence of nutrient concentration on investment into a pathway depends on the duration of nutrient availability. (**b**) Adaptive investment increases as the intervals between environmental switches increase. (**c**) Stochastic investment (*k*) in simulations with stochasticity enabled peaks near 0 and 1. (**d**) The plot shows the ratio of mean update steps between simulations with stochasticity enabled and simulations with stochasticity disabled (*time delay*). Simulations with disparate periods of nutrient availability need more update steps when stochasticity is enabled, while simulations with equal periods of availability require fewer update steps (exception: *d*_*i*_ = 1). (**e**) This disparity of mean update steps between simulations with stochasticity enabled and simulations with stochasticity disabled (*time delay*) decreases as generations increase.

Investment was focused on the nutrient of longer availability (Fig. 5a, Supplementary Fig. S9a), but with a much broader distribution than in the constant environment. Equal periods of availability led to equal investment. Environmental nutrient concentration became less influential on investment strategies as the frequency of environmental switches decreased. Investment was determined by nutrient concentration mainly when the environment switched at each time step (Fig. 5a). Adaptive investment increased as the frequency of environmental fluctuations decreased (Fig. 5b, Supplementary Fig. S9c). At intermediate switching frequencies, investment occurred roughly equally via the nutrient-dependent and constitutive pathway. Switching nutrients at every time step put importance on nutrient concentration again and investment occurred constitutively if nutrients were of equal concentration.

**Figure 6:**
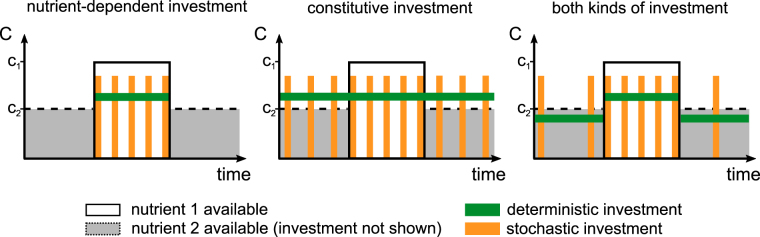
Nutrient-dependent and constitutive investment in relation to the presence of expression stochasticity. Nutrient-dependent investment occurs only in the presence of a nutrient, constitutive investment is independent of nutrient presence. The difference between deterministic and stochastic investment is in the temporal partitioning of resources. Deterministic investment leads to a continuous investment while stochastic investment produces occasional but more intense investments bursts. The frequency of burst depends on the relative metabolic rates.

Deterministic investment partitions the uptake into both nutrient pathways depending on the agents’ metabolic rates. Stochastic partitioning allocates all available uptake into one of the nutrient uptake pathways, choosing the pathway randomly with a probability depending on the agent’s metabolic rates. Stochastic investment in the deterministically alternating environment generally peaked near 0 and 1 (Fig. 5c). Contrary to the constant environment, because now both pathways received significant portions of general investment (Fig. 5a), this indicates two main kinds of stochastic investment strategies: i) Low stochastic investment caused most of the uptake to be invested deterministically. This way, both kinds of transporters were invested in and one of them got an additional boost, depending on the rate-dependent stochastic partitioning. ii) High stochastic investment caused most uptake to be invested stochastically. Now, only one of both uptake pathways received a large investment, and a small part was partitioned deterministically between both kinds of transporters. The difference between both strategies lies not in their total investment into one of the pathways as there are only small qualitative differences in the investment distributions of simulations with stochasticity enabled or disabled, but in the temporal partitioning of resources (Fig. 6). With an increase in total nutrient concentration, stochastic investment increased as well (Supplementary Fig. S9d). This suggests that at low nutrient concentrations a deterministic strategy is more advantageous compared to a stochastic strategy, and that at high nutrient concentrations, a stochastic strategy is either more advantageous or nutrient availability is sufficiently high to make stochastic investment tolerable.

To determine the effect of stochasticity on adaptation, we calculated the ratio of mean update steps between simulations with stochasticity enabled and simulations with stochasticity disabled (*time delay*). We found that the merit of stochastic gene expression depended on the periods of availability as well as nutrient concentration (Fig. 5d). Simulations with stochasticity enabled required significantly more update steps when nutrients were available for disparate periods of time or when switches happened at every update step (Supplementary Table). When nutrients were available for equal periods of time, simulations with stochasticity enabled required significantly less update steps compared to simulations with stochasticity disabled. In this case, the merit of expression stochasticity increased as nutrient concentrations increased. Thus, while non-stochastic expression was superior for disparate periods of nutrient availability or rapid switches between nutrients, stochastic expression was superior in environments with equal periods of availability. The dependence of the time delay on generation count reached (Fig. 5e) indicates that this is due to differences in the speed of adaptation in the presence of stochasticity. Together with our observation of only slight differences in investment strategies between simulations with stochasticity enabled and disabled (Fig. 5a,b) and a broader distribution of stochastic investment compared to in the constant environment, this indicates that stochastic investment can be used to fine-tune investment strategies in this environment.

#### Randomly Alternating Environments

**Figure 7:**
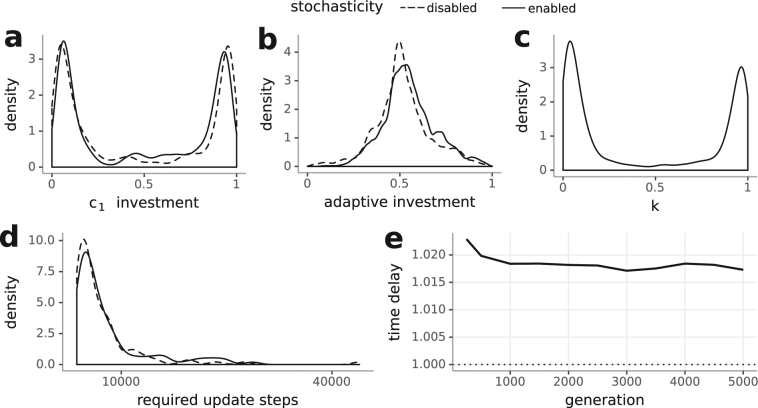
Randomly alternating environment: Investment strategies and required update steps. The legend at the top applies to (**a**–**d**). (**a**) Investment occurs into only one of the nutrients, (**b**) adaptive and constitutive investment are balanced and (**c**) stochastic investment (*k*) in simulations with stochasticity enabled peaks near 0 and 1. (**d**) Simulations with stochasticity enabled tend to require slightly more uptake steps than simulations with stochasticity disabled. (**e**) The ratio of mean update steps between simulations with stochasticity enabled and simulations with stochasticity disabled (*time delay* depends on the number of generations reached.

In the randomly alternating environments, investment strategies resembled those we found in the constant environment (Fig. 7a,b). Investment occurred both nutrient-dependent and constitutively and was focused on one nutrient. Stochastic investment peaked near 0 and 1 (Fig. 7c). However, *c*_1_ and stochastic investment were more broadly distributed. This suggests at least some degree of exploitation of stochasticity. Simulations with stochasticity enabled tended to require slightly more uptake steps than simulations with stochasticity disabled (Fig. 7d,e, Supplementary Table). This indicates that while stochasticity can sometimes be beneficial in the randomly alternating environment, it is detrimental in general.

## Discussion

To study the effect of the nutritional environment on optimal metabolic adaptation, including the optimal level of stochasticity, we combined an agent-based model of a minimal bacterial metabolism with an evolutionary algorithm. The model provides a wide spectrum of possible adaptations and we analyzed which parts of the solution space were occupied depending on different environmental conditions. We compared three nutritional environments that provide different challenges to their inhabiting organisms: The organisms needed to adapt to unchanging nutrient concentrations in the constant environment, to alternating but equal concentrations of a set duration of availability in the deterministically alternating environment, and to a range of possible concentrations and durations of which only extreme and mean values are known in the randomly alternating environment (Fig. 3). Real-life approximations of these abstract environments can be found for example in intracellular organisms (constant environment) or in the gut (deterministically alternating environments) or aquatic environments (randomly alternating environments).

How well organisms adapt to their environments depends on its predictability. Two aspects need to be combined: investment now and investment in the future. The constant environment allows fast and almost complete adaptation by specialization, barring the effects of mutation. In line with earlier findings^33,38^, stochasticity was detrimental and suppressed, but could, as in nature, not be totally eliminated. We found similar specialization in rapidly fluctuating environments. When environmental switches occurred within a generation (*d*_*i*_ = 1), it was advantageous to ignore short deviations^20,39^.

In a dynamic environment, it is of great importance whether investment occurs into a pathway whose corresponding environmental nutrient will still be available in the next time step or not. The agents cannot predict their environment, but they can anticipate changes using their evolved investment rates. Nutrient-dependent investment, representing gene expression dependent on an environmental cue, protracts the time it takes to respond to the occurrence of a nutrient, but it reduces investment into absent (or inferior) nutrients. Constitutive investment enables a fast response to the reappearance of a nutrient but entails wasting resources in its absence. Generally, the degree of adaptive investment depends on the value of a nutrient. Using nutrient-dependent and constitutive investment in equal measure provides a means of being ready to immediately use a nutrient upon occurrence and stocking up on transporters while it is available. Stochastic investment includes the benefit of fortuitous investment into a nutrient that is suddenly available due to an environmental switch, but also the cost of losing a full investment in its absence. At high nutrient concentrations individuals can tolerate a potential investment loss better than they can at low nutrient concentrations. Our results show only little qualitative differences in the adaptive investment distributions whether stochasticity was enabled or disabled, indicating a modulating effect of stochasticity. Adaptive investment can regulate the effect of stochastic investment by influencing the probability of investing into a pathway depending on the presence of the respective nutrient. Adaptive investment and supplemental stochastic investment are both viable strategies for surviving in an alternating environment. In nature, we can find examples of both strategies: there is positive selection for noise in genes whose optimal expression level is uncertain^15,16^ and there are transcription factors responding to environmental cues^18^.

Stochastic investment in the deterministically alternating environment peaks near 0 and 1 as in the constant environment, but is more broadly distributed. As *c*_1_ investment distributions are broader than in the constant environment, the probability of investing into transporters for an absent or lower concentrated nutrient is higher. In the case of high stochastic investment, one pathway regularly receives a large portion of available investment while the alternative one receives a large investment only rarely. We can interpret this as stochastic switching^3,20,21^.

Organisms in alternating environments need to be versatile and preserve a high degree of plasticity, and sometimes this can be achieved by exploiting inherent stochasticity. There are only small differences between the investment strategy distributions of environments with stochasticity enabled and disabled, yet there is a clear impact of stochasticity on performance. The ratio of mean update steps required to reach a preset mean number of generations between simulations with stochasticity enabled and simulations with stochasticity disabled (*time delay*) can be taken as a measure of the merit of stochasticity. In the constant environment stochasticity is always detrimental, except at equal nutrient concentrations. In the deterministically alternating environments, there is a clear dependence on the quality of fluctations: Stochasticity is only beneficial at equal periods of nutrient availability. In the randomly alternating environments, stochasticity is generally detrimental. Expressing transporters just in time (adaptive investment) risks not using a nutrient to its full potential but avoids squandering resources in anticipation of potential availability. Directing resources into a nutrient that is not available (stochastic investment) enables a fast reaction to environmental switches. With an increase in nutrient concentration in the deterministically alternating environment, the advantage of reacting faster compensates for the waste of nutrients. Stochasticity is beneficial above a threshold environmental richness; below, the penalty of being caught in an unfit state is too high^20^, but above it offers an advantage over responsive switching as total adaptation to the environment is impossible^17^ or too slow^3^. The *time delay* between generations was not constant (Figs 4e, 5e and 7e), indicating that stochasticity generally does not influence growth rates but the speed of adaptation. We found that stochasticity slowed down adaptation, except in equal periods of availability in the deterministically alternating environment.

Our findings lead us to expect similarities in the adaptation of organisms that inhabit environments with very frequent or wholly unpredictable changes between several nutrients (such as in aquatic environments) and organisms that live in environments that rarely change (such as endosymbionts), and a different set of adaptations in organisms that face environments that change regularly and predictably (such as in the human gut). Additionally, we expect organisms that evolved to live in constant environments to possess comparably lower noise levels in relevant functional gene groups (for example nutrient transporters in endosymbionts) compared to organisms that live in fluctuating environments, in which we found stochasticity to sometimes be advantageous.

Previous works have postulated that noise generally renders organisms both less adapted and less adaptable^40^ and is usually detrimental to fitness^41^. We do not find this to be true, rather, it depends on the circumstances. In essential and housekeeping genes noise is selected against^7,14^. In highly predictable environments, the genes that are left after genome reduction are all essential, which makes low noise in these genes a necessity. But in a fluctuating environment, essentiality depends on the exact environmental conditions at a specific moment and on the probable environmental changes in the near future. Resources must be directed both towards what is needed now and to what is potentially needed later. We have observed a clear advantage of stochasticity in some environmental conditions and a clear disadvantage in others. While stochasticity intrinsically introduces an element that hinders all individuals in a population from reaching an evolutionary optimal state and staying there, it enables a better adaptation of an isogenic population to fluctuating environments^38^.

We have investigated an agent-based model of a minimal bacterial metabolism to study the interactions between the nutritional environment and gene expression, both in the presence and absence of expression stochasticity. Our model is in accordance with previous findings and reveals that different environmental regimes can produce similar adaptations. We explored the interplay of nutrient-dependent and stochastic gene expression and found that nutrient-dependent investment is effective and sufficient for organisms to survive and reproduce successfully in fluctuating environments and that expression stochasticity is only of advantage if nutrients are of equal value to the organism. However, we did not present the organisms in our study with challenges as they occur in nature, like competition for resources, interaction with other individuals or environmental catastrophes. We demonstrated that investigating environment-noise interaction using agent-based modeling of generic bacteria is sensible. This is the first time that the environment-noise relationship has been investigated using this approach. Our model permits qualitative conclusions concerning the magnitude of stochasticity concerning the constrains of the environment, independent of its molecular origin. It allows the generalisation of our findings on nutritious environments to any environmental parameter that poses a clear signal which is recognized by the cell and turned into a reaction, and that has a strong impact on reproductive fitness – like the presence of antibiotics.

A main direction of future work is to investigate more complex environments, for example with both (or more) nutrients co-occurring and changing concentrations, approximating actual nutritional environments, and to broaden the framework to include other environmental factors. On the other hand, the bacterial metabolism can be extended to include longer and more complex pathways. Additionally, a comparative investigation of bacterial genomes regarding differences in noise levels or predicted noise levels based on the known genetic details of noise could shed more light on the environment-noise relationship in organisms with different types of habitats.

## Methods

### Model

The model description follows the ODD (Overview, Design concepts, Details) protocol^42^. Non-relevant paragraphs are omitted. We implemented the model in Netlogo 5.2.1^43^.

#### Purpose

The model simulates a simple bacterial metabolism which includes constitutively and transcriptionally regulated expression as well as gene expression stochasticity. We explore the trade-off between flexibility and efficiency of gene expression in the context of different environments by analyzing the evolutionary adjustment of gene expression to various environmental conditions, which are defined by the presence of nutrients over a period of time. We explore general regulatory patterns and focus on qualitative results.

#### Entities, state variables and scales

The environment contains the concentrations *c*_*i*_ of *n* nutrients and the respective durations *d*_*i*_ of their availability. We assume a homogeneous environment without concentration gradients.

Bacteria exist inside the environment. Each bacterium takes up nutrients via specific transporters *t*_*i*_. The regulatory network of each bacterium consists of a set of metabolic rates used to calculate investment into new transporters. Further variables associated with each bacterium are its size *s* representing biomass, an age *a* and a generation *g*. Parameters common to all bacteria are a maximum mutation step size *m* and the share of biomass *b* used for cell maintenance. We assume Michaelis-Menten kinetics.

All parameters and variables are unitless as the model aims only to capture qualitative behavior. A complete overview over state variables and parameters used is given by Table 1 and a bacterial agent is shown in Fig. 2. Nutrient uptake and investment are modeled as discrete events, and once the decision to divide is reached, cell-division happens instantaneously.

**Table 1:** State variables and parameters with their respective ranges of value.

Name	Description	Range	Type
*c*_*i*_	weight of concentration of nutrient *i*	[0.2]	environmental
*d*_*i*_	duration of *c*_*i*_	[0.100]	environmental
*t*_*i*_	amount of transporters for *c*_*i*_ uptake	${\mathbb{R}}\ge 0$	bacterial
*k*	share of nutrients taken up invested stochastically	[0.1]	bacterial
*cmr*_*det*,*i*_	constitutive metabolic rate for deterministic investment in *t*_*i*_	[0.1]	bacterial
*amr*_*det*,*i*_	nutrient-dependent metabolic rate for deterministic investment in *t*_*i*_	[0.1]	bacterial
*cmr*_*stoch*,*i*_	constitutive metabolic rate for stochastic investment in *t*_*i*_	[0.1]	bacterial
*amr*_*stoch*,*i*_	nutrient-dependent metabolic rate for stochastic investment in *t*_*i*_	[0.1]	bacterial
*s*	size	${\mathbb{N}}$	bacterial
*a*	age, number of update steps since division	${\mathbb{N}}$	bacterial
*g*	generation, number of divisions experienced	${\mathbb{N}}$	bacterial
*m*	maximum mutation step size	0.01	global
*K*_*M*,*i*_	Michaelis-Menten constant	1	global
*K*_*cat*,*i*_	efficiency of catalysators	1	global
*b*	share of biomass used for maintenance	0.05	global

We implemented the model with *i* = 2. Environmental and global parameters are fixed at the beginning of each simulation run, bacterial parameters differ between individuals and change in the course of the simulation.

#### Process overview and scheduling

An update step includes, in that order, bacterial nutrient uptake, investment into new transporters, growth due to uptake, division and death, possible dilution of the bacterial population and an update of the environment (see submodels). Bacterial updates take place synchronously as they do not depend on the states of other bacteria. Simulations stop when all bacteria are dead or a preset mean number of generations has been reached.

#### Design concepts

Basic principles. The growth and life cycle of bacterial cells which can grow on two nutrients delivered by the environment is simulated and embedded into an evolutionary algorithm to allow adaptation to a specific environment. The evolutionary algorithm accomplishes the evolutionary adaption of uptake rates by minimizing the error (i.e. *e*_1_ and *e*_2_) of improper investment (see Fig. 1).

Bacteria take up nutrients depending on their concentrations and the presence of the respective bacterial transporters. The growth of a bacterium and investment into transporters for nutrient uptake is determined by metabolic rates. Nutrient uptake is altered in the next update step due to a changed number of transporters.

The amount of combined nutrients invested in additional transporters is determined by a regulatory network that consists of a set of constitutive and nutrient-dependent rates. Nutrient-dependent metabolic rates are nutrient-sensitive and correspond to transcriptionally regulated (substrate-dependent) expression, implying an underlying sensing machinery. Constitutive metabolic rates correspond to constitutive expression.

To model stochastic expression, a part *k* of the nutrients is stochastically invested. *k* thus represents the degree of deflection of expression control to encounter the uncertainties of a fluctuating environment (see Fig. 1). To separate stochastic and deterministic expression, each of these pathways has a separate set of nutrient-dependent and constitutive metabolic rates: For each pathway *i* there are four metabolic rates (see Table 1). This allows both deterministic and stochastic investment to be independently dependent on nutrient availability. The stochastic pathway is intended to combine the effects of transcriptional and translational bursting. To disable stochastic investment, *k* can be initialized as and kept at 0.

Cell division happens at a threshold size. The less time there is between cell division events in a bacterial lineage, the more frequent its genetic metabolic setup appears in the population, with small variations.

Emergence. The model selects for the most suitable metabolic setups (metabolic investment rates *cmr*_*det*,*i*_, *amr*_*det*,*i*_, *cmr*_*stoch*,*i*_, *amr*_*stoch*,*i*_ and *k*) in a given environmental regime by minimizing the investment in an improper uptake path (i.e. error *e*_1_ and *e*_2_). These metabolic solutions to a specific environment emerge over time as a consequence of the constraints of selection exerted by the evolutionary algorithm.

Stochasticity. The metabolic rates of the bacteria are randomly initiated following a uniform distribution. Whether investment via the stochastic pathway occurs into the first or the second nutrient is decided stochastically (see submodel Investment). The probability of cell division is linearly dependent on the size of the bacterium. Metabolic rates of daughter cells are mutated. Mutations are drawn from a symmetric triangular distribution whose limits are determined by the maximum mutation step size. In the randomly alternating environment the duration of availability and the nutrient concentration are drawn from a uniform distribution.

#### Initialization

The model offers three environments characterized by the duration of availability of food sources and their concentration. Nutrient concentrations *c*_*i*_ and their respective periods of availability *d*_*i*_ can be chosen manually. Metabolic rates (including *k*) for each bacterium are each drawn from a uniform distribution between 0 and 1. It is possible to switch the stochastic pathway off by forcing *k* = 0 (“stochasticity is disabled”). *K*_*M*,*i*_ and *K*_*cat*,*i*_ are each set to 1 and the maximum mutation step size *m* to 0.01. The amount of biomass used for maintenance is arbitrarily chosen as *b* = 5%. Thus, at each simulation step biomass and transporter numbers are reduced by 5%. Each bacterium starts with an initial size *s* = 1 and *t*_*i*_ = 1 transporter for each nutrient pathway *i*. The simulation starts with 500 bacteria and when the population reaches a threshold size of 4000 individuals, 70% of the individuals are randomly removed. Different values revealed no qualitative differences in the final investment values (Supplementary Figs S1, S2 and S3).

#### Input data

The model does not use input data to represent time-varying processes.

#### Modeled processes (submodels)

Nutrient uptake, investment, growth, division and death are, in that order, each executed by every bacterium. Dilution and the update of the environment are global processes.

Nutrient uptake. First, the nutrient uptake *u*_*i*_ via the respective pathway *i* is calculated following Michaelis-Menten kinetics with parameters *K*_*M*,*i*_ and *K*_*cat*,*i*_:

1 $${u}_{i}=\frac{{t}_{i}\cdot {K}_{cat,i}\cdot {c}_{i}}{{K}_{M,i}+{c}_{i}}$$

*t*_*i*_ is the amount of transporters and *c*_*i*_ the concentration of the respective nutrient in the environment. If the calculated uptake exceeds *c*_*i*_, *u*_*i*_ is set to *c*_*i*_. The combined uptake of all nutrients amounts to

2 $$u=\sum _{i}\,{u}_{i}\mathrm{.}$$

Investment. The share of nutrients invested via the stochastic pathway is *k*, thus the total amount of nutrients invested via the stochastic pathway amounts to

3 $${u}_{stoch}=u\cdot k$$

and the total amount invested via the deterministic pathway amounts to

4 $${u}_{det}=u\cdot (1-k)=u-{u}_{stoch}\mathrm{.}$$

Investment via the deterministic pathway uses the rates *cmr*_*det*,*i*_ and *amr*_*det*,*i*_, investment via the stochastic pathway uses the rates *cmr*_*stoch*,*i*_ and *amr*_*stoch*,*i*_. The relative amount of nutrients invested via the deterministic pathway into transporters for each nutrient *i* is calculated by

5 $${n}_{det,i}=\frac{cm{r}_{det,i}+am{r}_{det,i}\cdot {c}_{i}}{{\sum }_{j}(cm{r}_{det,j}+am{r}_{det,j}\cdot {c}_{j})}.$$

Thus the increase of transporters for each pathway *i* via the deterministic pathway amounts to

6 $${\rm{\Delta }}{t}_{det,i}={n}_{det,i}\cdot {u}_{det}.$$

The share of nutrients invested via the stochastic pathway is allocated randomly with a rate-dependent probability (all-or-nothing). The probability of investment into pathway *i* is

7 $${n}_{stoch,i}=\frac{cm{r}_{stoch,i}+am{r}_{stoch,i}\cdot {c}_{i}}{{\sum }_{j}(cm{r}_{stoch,j}+am{r}_{stoch,j}\cdot {c}_{j})}\mathrm{.}$$

A random number 0 ≤ *p* ≤ 1 is drawn from a uniform distribution. *n*_*stoch*_ is fully invested into pathway *i* if

8 $$p\in [\sum _{q=0}^{i-1}\,{n}_{stoch,i},\sum _{q=0}^{i}\,{n}_{stoch,i}].$$

Thus

9 $${\rm{\Delta }}{t}_{stoch,i}={n}_{stoch,i}\cdot {u}_{stoch}$$

for the chosen pathway *i* and Δ*t*_*stoch*,*i*_ = 0 for all others. The number of transporters for each pathway is subsequently updated:

10 $${t}_{i}^{new}=({t}_{i}^{old}+{\rm{\Delta }}{t}_{stoch,i}+{\rm{\Delta }}{t}_{det,i})\cdot (1-b).$$

Growth. Bacterial age increases by 1 and the size *s* is updated according to

11 $${s}^{new}=({s}^{old}+u)\cdot \mathrm{(1}-b\mathrm{).}$$

Division. The probability of cell division is

12 $$p=\,{\rm{\max }}\,\{0,\,{\rm{\min }}\,\{1,s-2\}\}$$

Parent and daughter each receive half of the transporters, their size is set to half the parent’s size and both ages are reset to 0. The metabolic rates and *k* of the daughter cell are each mutated according to a triangular distribution:

13 $$valu{e}^{daughter}=valu{e}^{parent}+({v}_{1}-{v}_{2})$$

with ${v}_{1},\,{v}_{2}\in {\mathbb{R}}$ randomly drawn from a uniform distribution between 0 and the maximum mutation step size *m*. The generation count of both cells increases by 1.

Death. If the size of a bacterium drops below 0.5, it dies. 0.5 is half the original size and can only be reached when nutrient uptake has been insufficient to balance maintenance costs for more than 10 update steps. The bacterium’s resources are removed from the system.

Dilution and Update Environment. If the bacterial count exceeds 4000 individuals, the population is diluted by 70 per cent. Dilution takes individuals out of the simulation irrespective of their genotype, creating genetic drift and keeping the population size computationally manageable.

If a constant food environment is chosen, no further action is necessary. If an alternating environment is chosen, the update proceeds as follows. At any time, only one nutrient is available (*c*_*i*_ > 0) and the other’s concentration is set to 0. Each nutrient is available for *d*_*i*_ update steps. In the randomly alternating environment, *d*_*i*_ is drawn from a uniform distribution between 1 and 50. If *d*_*i*_ of the focal nutrient is reached, *c*_*i*_ is set to 0. In the deterministically alternating environment, the other nutrient’s concentration is set to and kept at its preset value. In the randomly alternating environment, a value from a uniform distribution between 0.1 and 2 is drawn at each update step. Thus, only one nutrient at a time is available.

### Analysis

After the simulation stops, we calculated *c*_1_ investment and adaptive investment for each individual by:

14 $${c}_{{\rm{1}}}\,{\rm{investment}}=(1-k)\cdot \tfrac{cm{r}_{det\mathrm{,1}}+am{r}_{det\mathrm{,1}}}{{\sum }_{i}(cm{r}_{det,i}+am{r}_{det,i})}+k\cdot \tfrac{cm{r}_{stoch\mathrm{,1}}+am{r}_{stoch\mathrm{,1}}}{{\sum }_{i}(cm{r}_{stoch,i}+am{r}_{stoch,i})}$$

15 $${\rm{adaptive}}\,{\rm{investment}}=(1-k)\cdot \tfrac{{\sum }_{i}am{r}_{det,i}}{{\sum }_{i}(cm{r}_{det,i}+am{r}_{det,i})}+k\cdot \tfrac{{\sum }_{i}am{r}_{stoch,i}}{{\sum }_{i}(cm{r}_{stoch,i}+am{r}_{stoch,i})}$$

All analyses were performed using the R software version 3.0.2^44^. All plots show the calculated investment rates based on the pooled calculated investment of all individuals across all applicable conditions, combining individuals from separate simulation runs. Update steps required to reach a preset number of generations were compared with Mann–Whitney-U-tests. The false discovery rate (FDR) procedure of Benjamini *et al*.^45^ was applied to correct p-values after multiple testing.

## Electronic supplementary material

Supplementary Table

Supplementary Information
