# Supplementary material for: Computational Investigation of Environment-Noise Interaction in Single-Cell Organisms: The Merit of Expression Stochasticity Depends on the Quality of Environmental Fluctuations
Source: Sci Rep. 2018 Jan 10;8:333. doi: 10.1038/s41598-017-17441-8 (PMC5762857; doi:10.1038/s41598-017-17441-8)
Supplement: Supplementary file 2 — Supplementary Information [file 41598_2017_17441_MOESM2_ESM.pdf]

Computational Investigation of Environment-Noise Interaction in Single-Cell Organisms: The Merit of Expression Stochasticity Depends on the Quality of Environmental Fluctuations

Anja Lück (1), Lukas Klimmasch (2), Peter Großmann (1), Sebastian Germerodt (1,+), and Christoph Kaleta (3,+,*)

1 Department of Bioinformatics, Friedrich Schiller University, Jena, 07743, Germany
2 Group Theoretical Systems Biology, Friedrich Schiller University, Jena, 07743, Germany
3 Research Group Medical Systems Biology, Institute for Experimental Medicine, Christian-Albrechts-University, Kiel, 24105, Germany

* c.kaleta@iem.uni-kiel.de
+ these authors contributed equally to this work

Supplementary Fig. S1

Sensitivity analysis: constant environment.

The plots show c_1 investment, adaptive investment and stochastic investment as they depend on the number of bacterial generations until the simulation is stopped, initial number of individuals, dilution rates and the population size at which dilution occurs. Lines indicate the respective Loess curves of the repeated simulations' (n = 100) respective means and shaded areas indicate the respective 95% confidence intervals. Legends apply row-wise. Colors for stochastic investment (3rd row) indicate combinations of concentrations (c_1-c_2). General and relative adaptive investment do not change with changes in the initial parameters and relative stochastic investment rates show minor qualitative changes.

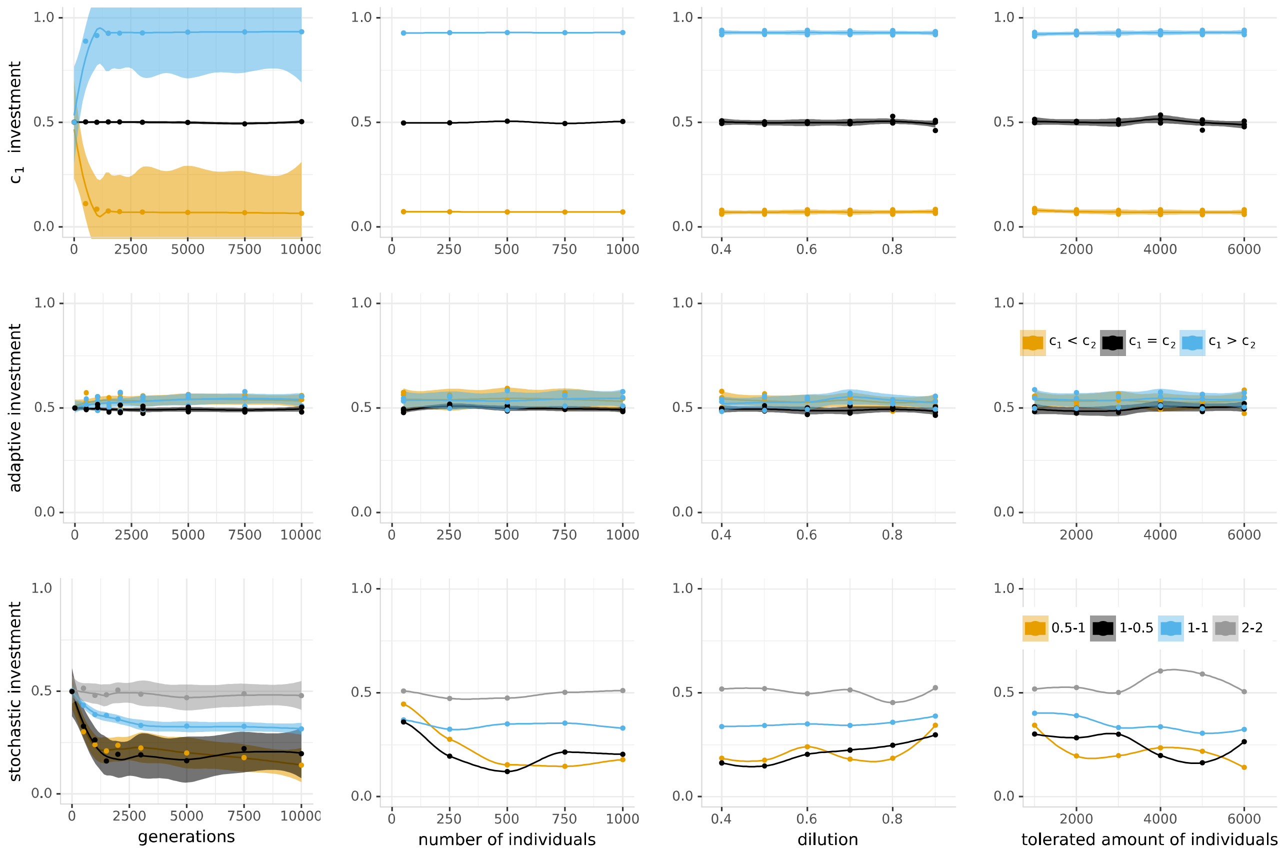

Supplementary Fig. S2

Sensitivity analysis: deterministically alternating environment.

The plots show c_1 investment, adaptive investment and stochastic investment as they depend on the number of bacterial generations until the simulation is stopped, initial number of individuals, dilution rates and the population size at which dilution occurs. Lines indicate the respective Loess curves of the repeated simulations' (n = 100) respective means and shaded areas indicate the respective 95% confidence intervals. Legends apply row-wise. Colors for stocvhastic investment (3rd row) indicate mean duration of availability ($(d_1+d_2)/2$). General and relative adaptive investment do not change with changes in the initial parameters and relative stochastic investment rates show minor qualitative changes.

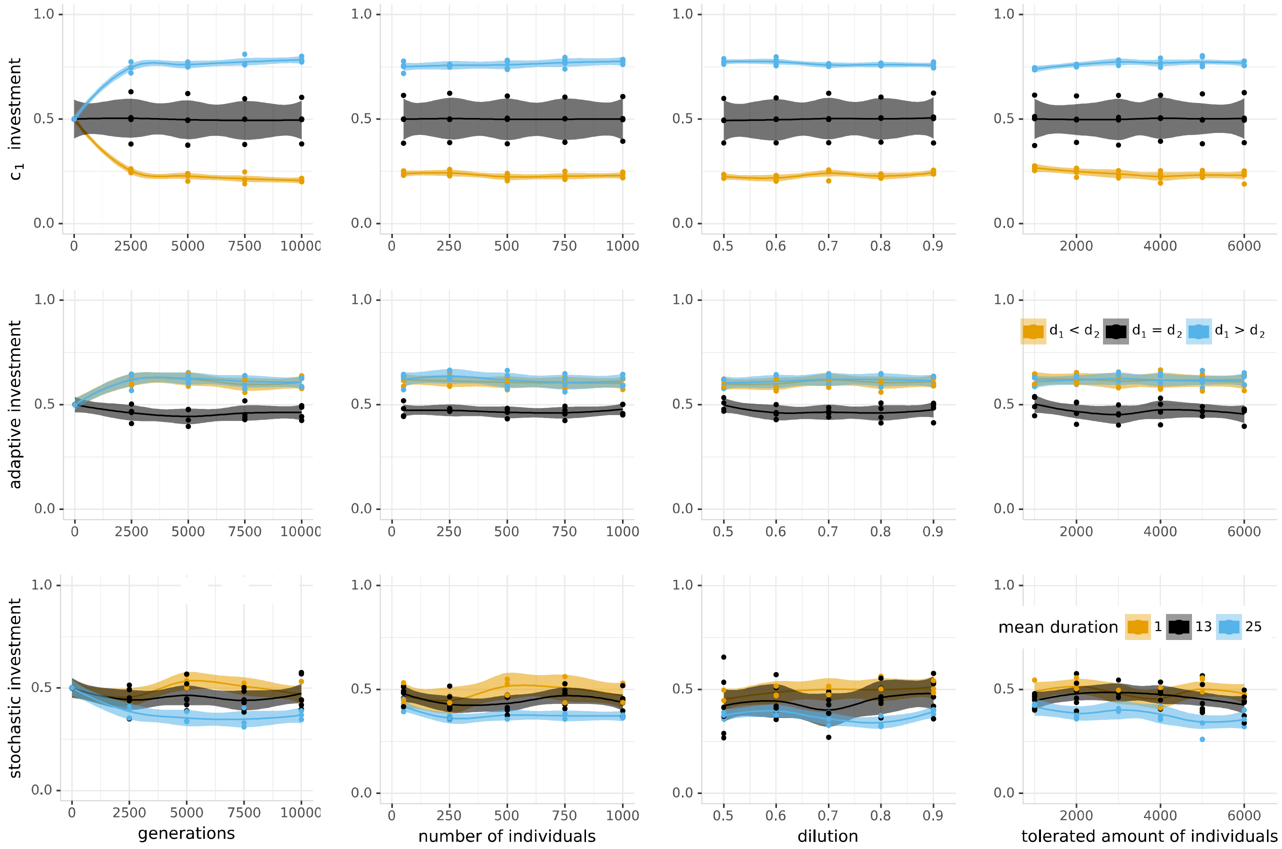

Supplementary Fig. S3

Sensitivity analysis: randomly alternating environment.

The plots show c_1 investment, adaptive investment and stochastic investment as they depend on the number of bacterial generations until the simulation is stopped, initial number of individuals, dilution rates and the population size at which dilution occurs. Lines indicate the respective Loess curves of the repeated simulations' (n = 100) respective means and shaded areas indicate the respective 95% confidence intervals. The respective investment rates show merely minor qualitative changes.

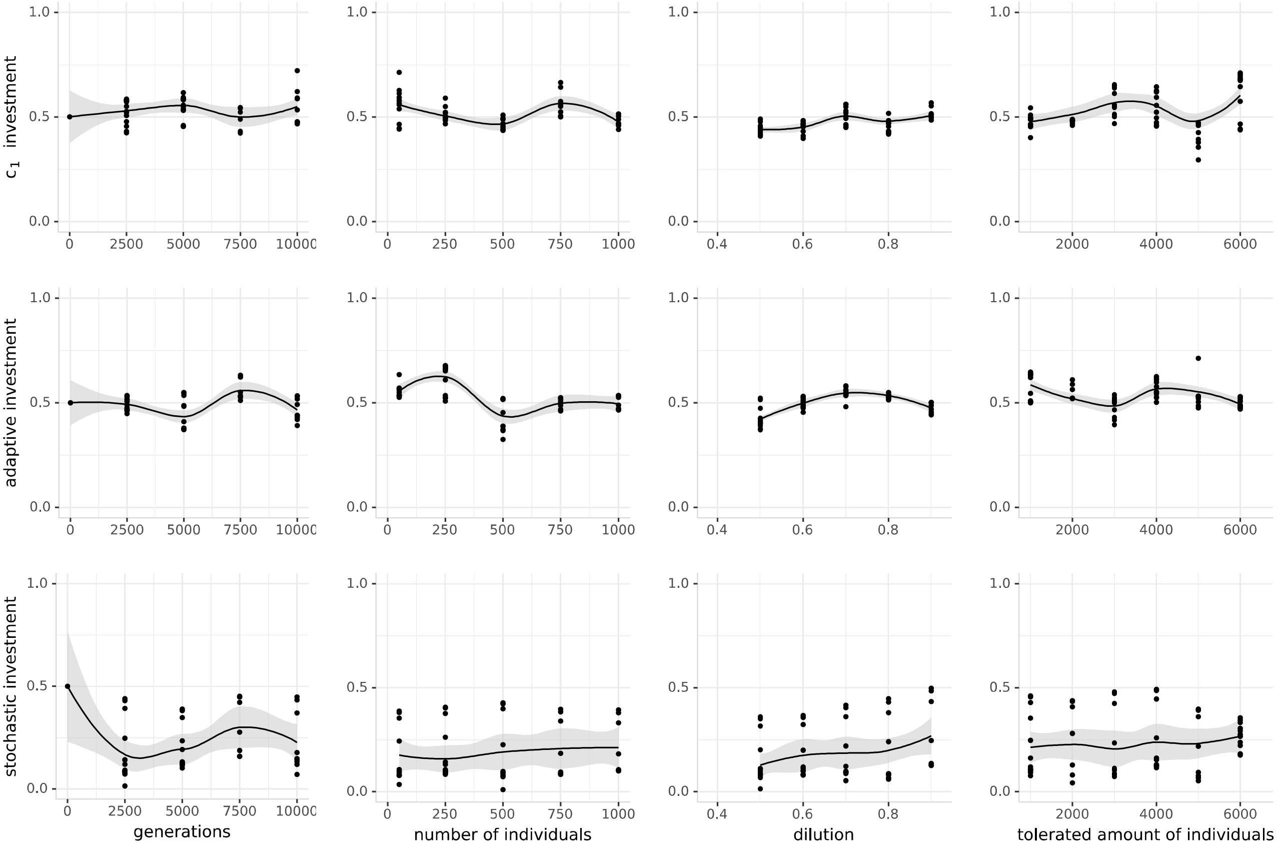

Supplementary Fig. S4
Constant environment: Density of c_1 investment, adaptive investment and stochastic investment.
Investment is focused on the nutrient available in the higher concentration. Adaptive and constitutive investment are balanced. For equal concentrations, the focus is in more deterministic investment, and in the other cases investment happens either deterministically or stochastically.

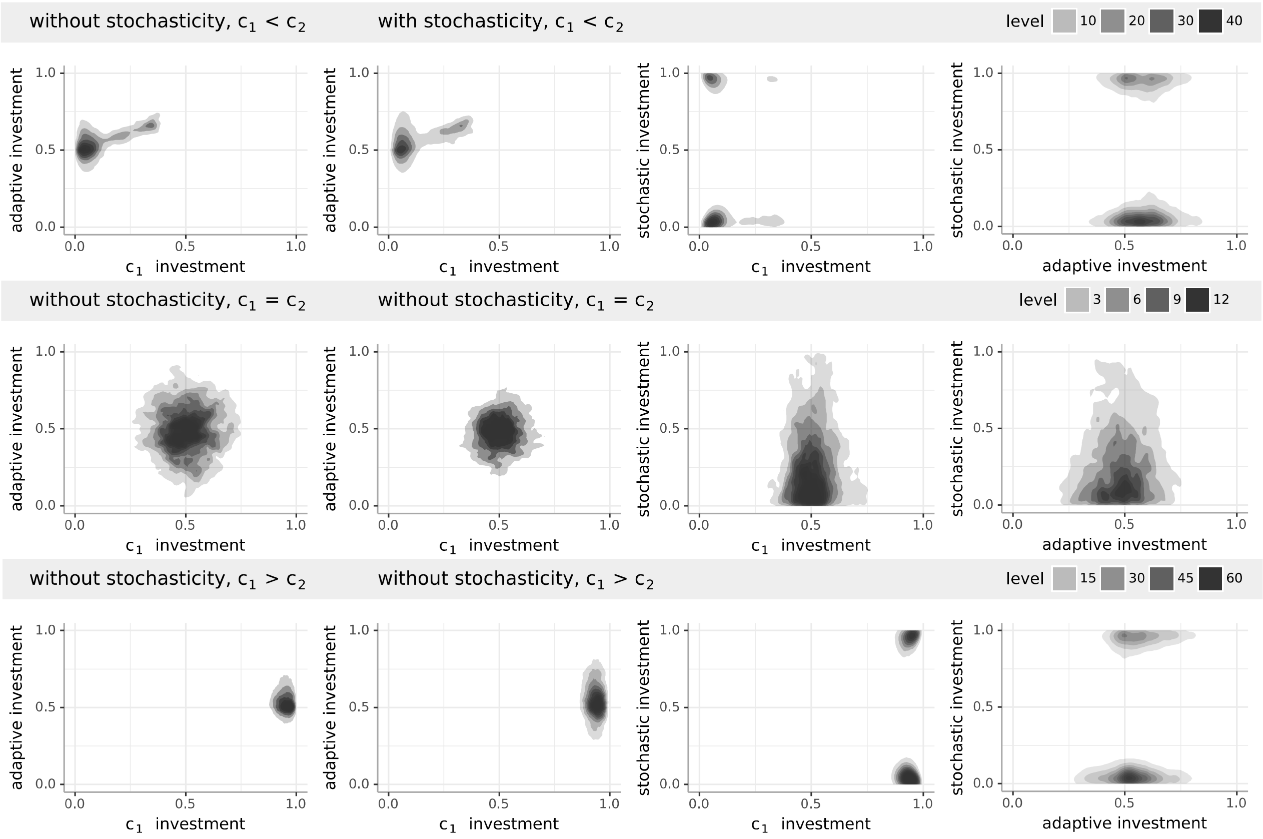

Supplementary Fig. S5

Constant environment: Investment trends relative to the total nutrient concentration available.

a c_1 investment is focused on the nutrient available in the higher concentration.

b Adaptive investment generally takes on middle values. **c** Stochastic investment increases as total nutrient concentration increases.

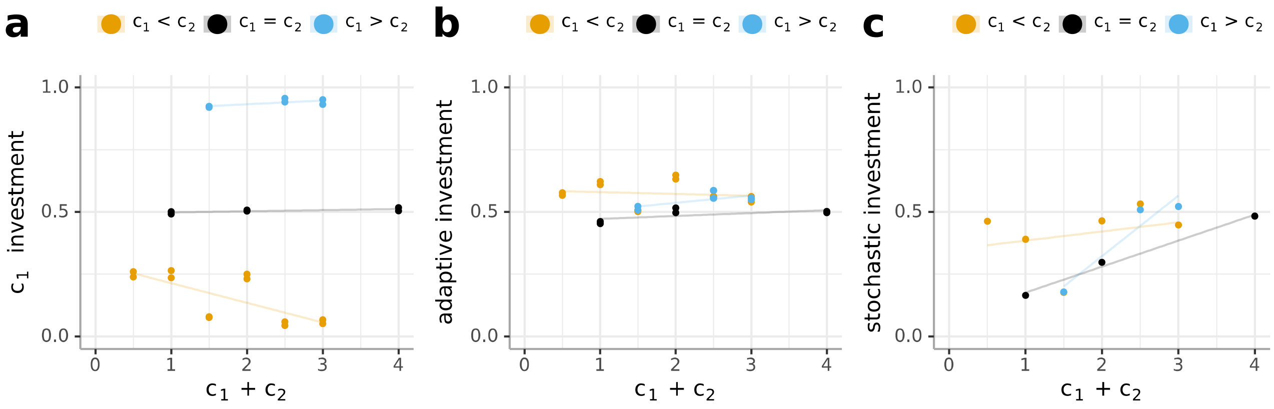

Supplementary Fig. S6
Constant environment: Required update steps in relation to the total nutrient concentration available.
As concentrations increase, less update steps are necessary to reach 5000 generations. If nutrient concentrations are disparate, simulations need fewer steps than when nutrient concentrations are equal.

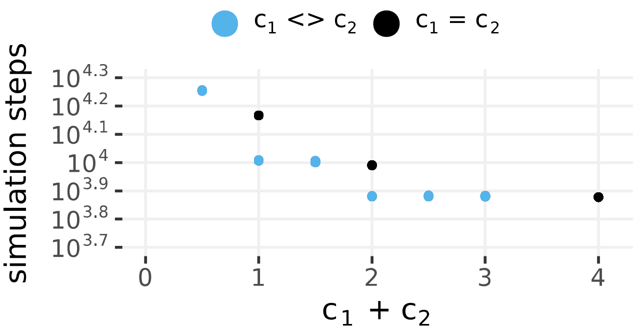

Supplementary Fig. S7

Constant environment: age distribution.

Horizontal panels indicate c_1 concentrations, vertical ones c_2 concentrations. X-axis denotes individual age. Age is reset to 0 after each division. Low nutrient concentrations lead to later division compared to higher nutrient concentrations.

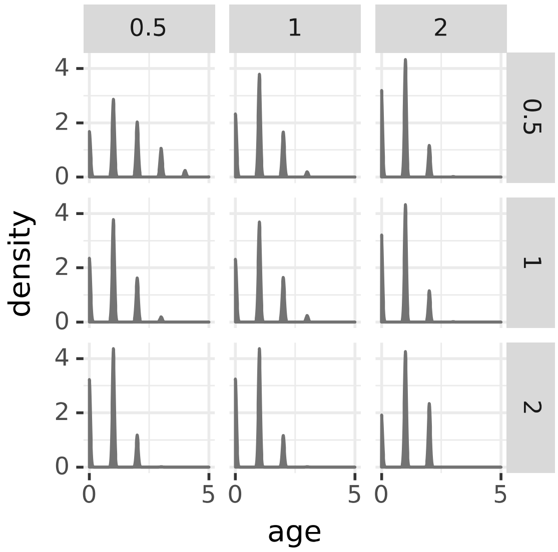

Supplementary Fig. S8
Deterministically alternating environment: age distributions.
X-axis denotes individual age. Age is reset to 0 after each division. Low total nutrient concentrations (rows) lead to later division compared to higher nutrient concentrations.

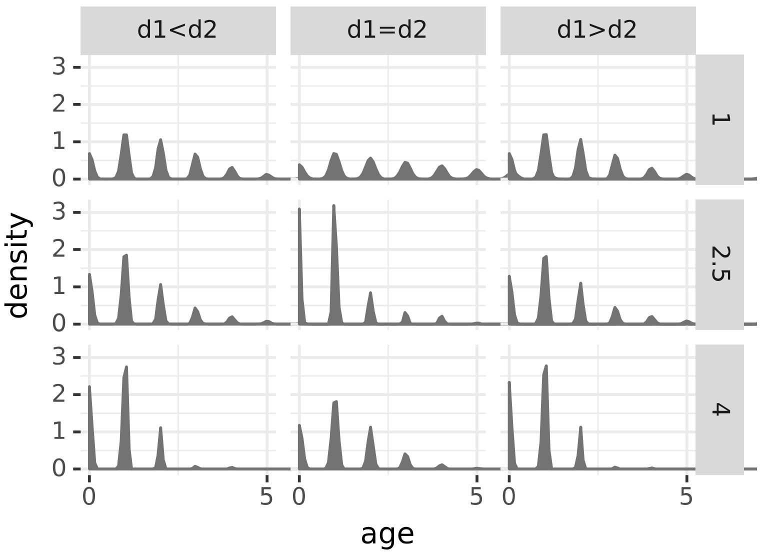

Supplementary Fig. S9

Deterministically alternating environment: Investment trends relative to the total nutrient concentration. And mean duration of availability.

a c_1 investment is dependent on the period of availability, but not on nutrient condcentration. **b, c** Adaptive investment is independent of nutrient concentration and increases as mean period of availability/absence of the nutrients increases. **d** Stochastic investment increases as nutrient concentrations increase. **e** The number of update steps required to reach 5000 generations decreases as nutrient concentrations increase. At equal nutrient concentrations simulations with lower switch frequency need fewer steps than simulations with higher switch frequency.

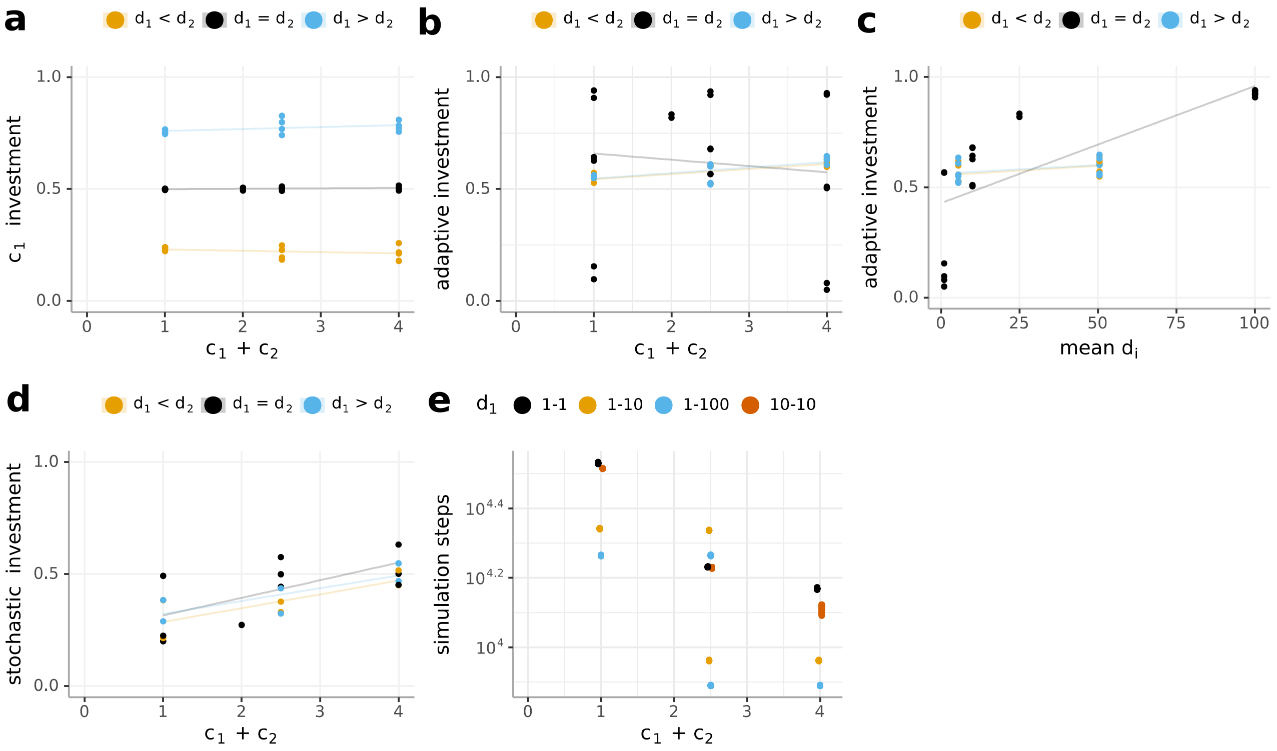

Supplementary Fig. S10
Deterministically alternating environment: Density of c_1 investment, adaptive investment and stochastic investment dependent on period of nutrient availability. Investment is focused on the nutrient that is available for longer periods of time. Adaptive and constitutive investment are balanced. Stochastic investment in is either high or low.

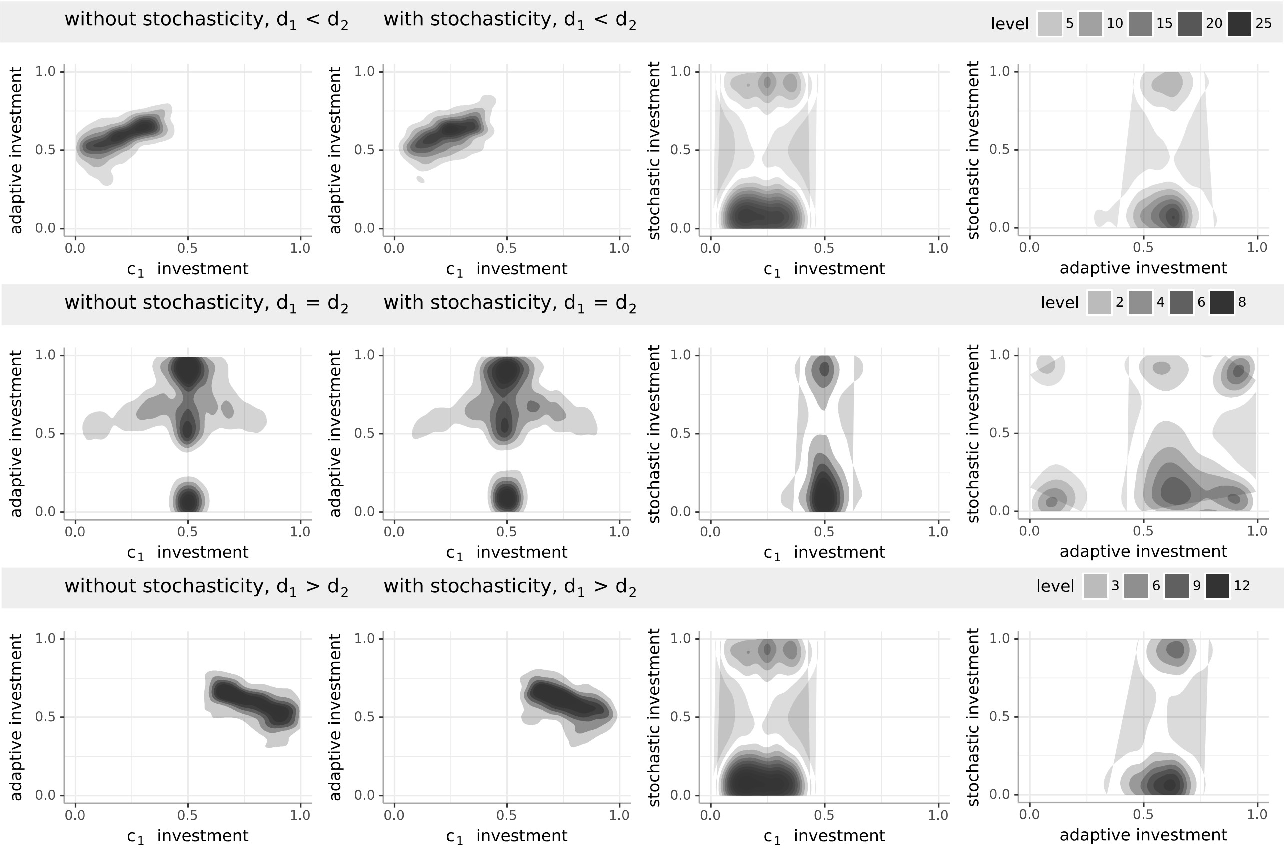

Supplementary Fig. S11

Deterministically alternating environment: Density of c_1 investment, adaptive investment and stochastic investment dependent on nutrient concentration.

In contrast to period of nutrient availability, nutrient concentration affects investment strategies less intensely.

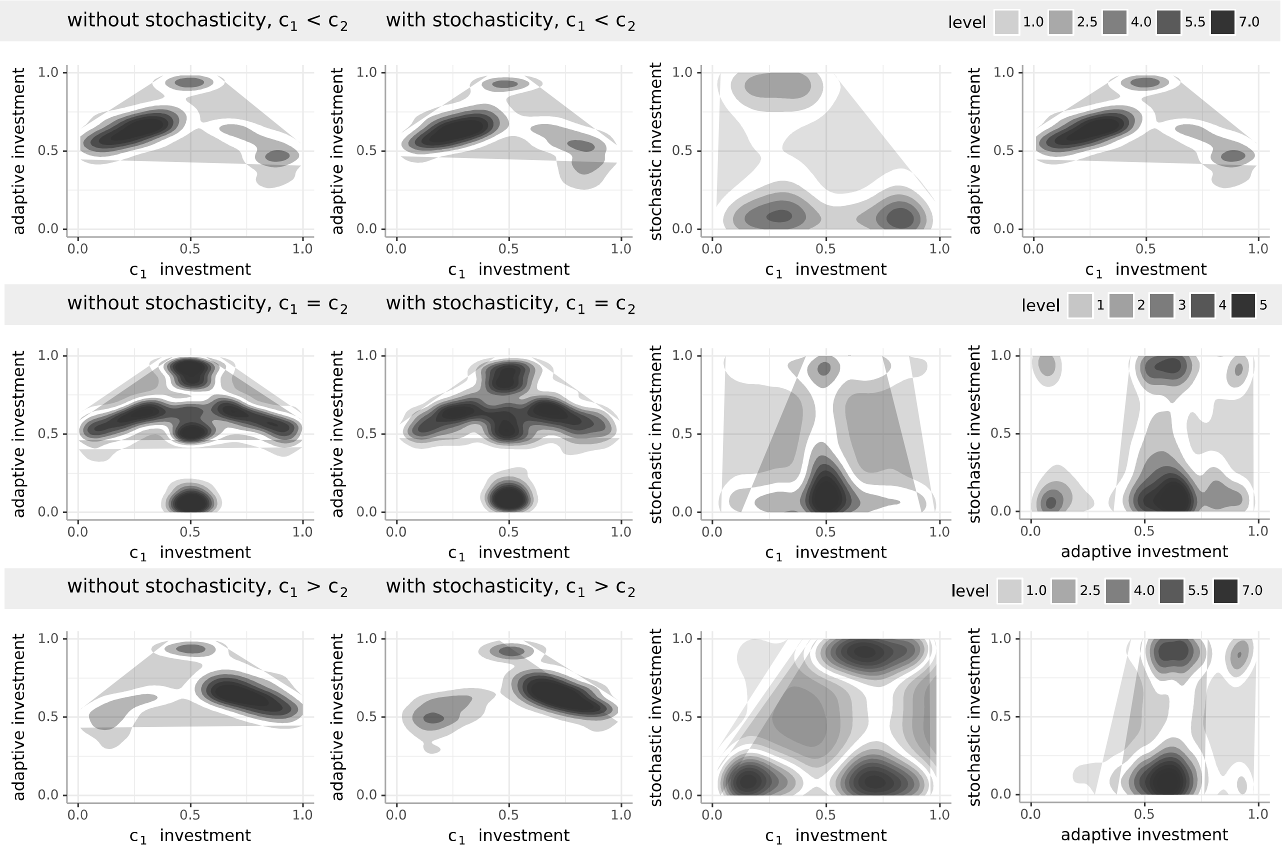

Supplementary Fig. S12

Randomly alternating environment: Density of c_1 investment, adaptive investment and stochastic investment.

Investment is focused on one nutrient and occurs both adaptively and constitutively. Stochastic investment peaks near 0 and 1.

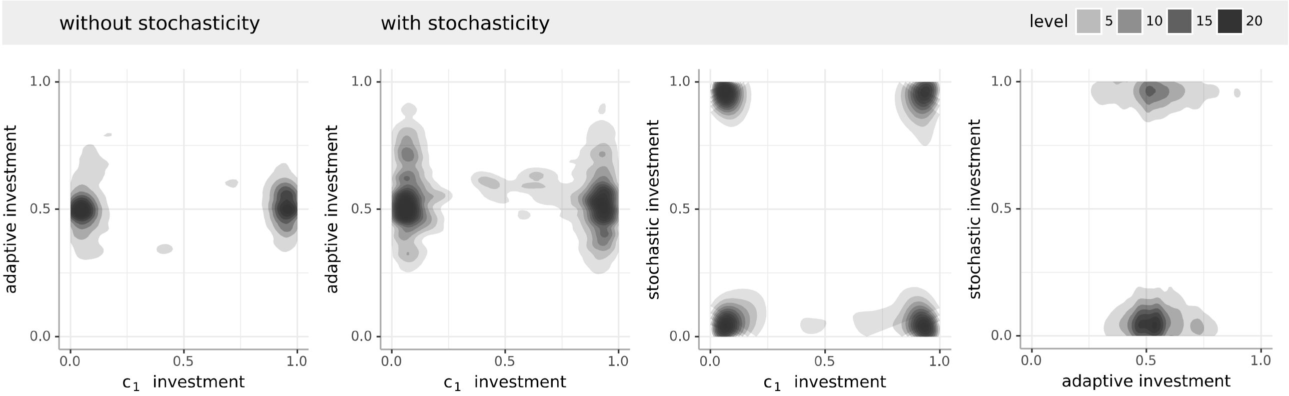

Supplementary Fig. S13
Environmental parameter combinations.
Simulations were run 100 times for each parameter combination. Across these 100 simulations, all surviving individuals were pooled in the analysis.

Environment	c_1	c_2	d_1	d_2	Total individuals	Mean required update steps: stochasticity disabled (standard deviation)	Mean required update steps: stochasticity enabled (standard deviation)
**constant**	0.5	0.5	-	-	440080	14665.22 (2.44)	14669.07 (5.74)
	0.5	1	-	-	464782	10019.74 (6.54)	10052.58 (36.62)
	0.5	2	-	-	446123	7593.71 (8.24)	7618.40 (13.43)
	1	0.5	-	-	453629	10021.47 (6.85)	10052.55 (32.76)
	1	1	-	-	436251	9785.56 (0.94)	9786.63 (2.11)
	1	2	-	-	444617	7584.34 (7.04)	7605.87 (11.66)
	2	0.5	-	-	461782	7592.91 (6.92)	7617.35 (13.30)
	2	1	-	-	461471	7583.78 (7.13)	7606.58 (12.03)
	2	2	-	-	491204	7542 (0)	7542 (0)
**Deterministically alternating**	0.5	0.5	1	1	442994	33806.09 (49.90)	33888.98 (72.20)
	0.5	0.5	1	10	455507	21935.25 (21.34)	21968.06 (27.07)
	0.5	0.5	1	100	473108	18360.57 (21.36)	18402.01 (27.49)
	0.5	0.5	10	1	455756	21930.29 (21.43)	21960.53 (27.81)
	0.5	0.5	10	10	466316	32719.33 (22.95)	32700.11 (33.47)
	0.5	0.5	100	1	452503	18361.02 (22.41)	18402.09 (27.17)
	0.5	0.5	100	100	324032	20682.57 (31.60)	20654.85 (63.16)
	0.5	2	1	1	451254	17024.50 (9.75)	17045.49 (16.11)
	0.5	2	1	10	479508	9148.03 (5.62)	9161.20 (12.65)
	0.5	2	1	100	471194	7755.87 (5.81)	7765.26 (9.88)
	0.5	2	10	1	469062	21697.87 (20.66)	21717.19 (31.54)
	0.5	2	10	10	459867	16997.70 (7.46)	16972.52 (34.74)
	0.5	2	100	1	458357	18355.22 (20.75)	18392.07 (27.10)
	0.5	2	100	100	270131	11983.20 (8.97)	11936.24 (68.67)
	2	0.5	1	1	465392	17027.34 (11.57)	17043.53 (15.70)
	2	0.5	1	10	463970	21697.38 (21.17)	21713.93 (24.64)
	2	0.5	1	100	427751	18357.34 (19.46)	18394.82 (29.86)
	2	0.5	10	1	455107	9146.29 (6.47)	9157.98 (10.70)
	2	0.5	10	10	458270	16976.06 (7.56)	16946.21 (34.74)
	2	0.5	100	1	475682	7751.36 (5.52)	7761.44 (9.60)
	2	0.5	100	100	294225	11860.70 (10.59)	11792.78 (84.93)
	2	2	1	1	422601	14746.77 (20.71)	14760.87 (50.91)
	2	2	1	10	449441	9145.52 (5.42)	9161.38 (12.78)
	2	2	1	100	443704	7754.48 (5.46)	7766.92 (10.89)
	2	2	10	1	449724	9143.30 (5.98)	9156.18 (9.56)
	2	2	10	10	457026	13254.50 (6.05)	12981.83 (237.12)
	2	2	100	1	454684	7750.65 (4.50)	7761.37 (8.78)
	2	2	100	100	295858	8397.02 (13.89)	8309.14 (99.41)
	1	1	25	25	448155	14766.42 (9.38)	14663.81 (10.62)
**Randomly alternating**	-	-	-	-	461856	9258.05 (4518.28)	9427.44 (3067.53)
